# Solvent-Free Cold Plasma Deposition of PVA–Antibiotic Films: Influence of Process Parameters on Coating Structure and Drug Release

**DOI:** 10.3390/polym18151839

**Published:** 2026-07-27

**Authors:** Abdugafarova Kibriyanur, Berillo Dmitriy, Zulyarov Samrat, Mohammad Kamran Saba, Dias Tastanbekov, Dmitry Rychkov

**Affiliations:** 1 The Institute of Computational Chemistry and Engineering, Tole Bi 160, Office 84, Almaty 050069, Kazakhstan; 2 Department of Science and Technical Development, M. Kozybayev North-Kazakhstan University, Zhumabaeva Street 114, Petropavlovsk 150000, Kazakhstan; daberillo@ku.edu.kz; 3 Athletes’ Anti-Doping Laboratory, Gagarin Avenue 50, Almaty 050009, Kazakhstan; 4 Higher School of Medicine, Al-Farabi Kazakh National University, Tole Bi Street 96, Almaty 050012, Kazakhstan; 5 Chair of Chemical & Biochemical Engineering, Satbayev University, Almaty 050013, Kazakhstan; 6 Qazaqstan Advanced Institute of Science and Technology, Project Office, Satpayev Str. 29/6, Almaty 050040, Kazakhstan; 7 “Akyldy Food Supplements” Ltd., Auezov Street 145v, Almaty 050057, Kazakhstan; 8 Technology Transfer Center Weissenburg, Faculty of Engineering, Ansbach University of Applied Sciences, Richard-Stücklen-Straße 3, 91781 Weißenburg in Bayern, Germany; dmitry.rychkov@hs-ansbach.de

**Keywords:** cold atmospheric plasma, polyvinyl alcohol, amikacin release, medical implants, drug release kinetics, sustained release

## Abstract

Post-operative infections remain a challenge in implant surgery, leading to prolonged treatment, increased costs, and implant failure. Localized antibiotic-delivery coatings are a promising strategy to reduce infection risk while maintaining biocompatibility. Here, we evaluate cold atmospheric pressure plasma (CAP) spraying as a solvent-free method to deposit polyvinyl alcohol (PVA) layers containing amikacin on stainless steel and to identify plasma parameters that control release and antibacterial activity. A 3 × 3 factorial design varied nozzle distance (15, 20, 25 mm) and speed (10, 15, 20 cm/s). Surface morphology was assessed by optical microscopy, amikacin release quantified by HPLC-HRMS, and antibacterial activity tested against *Staphylococcus aureus* and *Escherichia coli* using Kirby–Bauer disc diffusion. Two-way ANOVA with Tukey post hoc tests and nonparametric validation were applied. Cold plasma spraying speed significantly affected drug release, whereas distance and the interaction term were not significant. Lower spraying speeds produced thicker, more porous coatings with greater cumulative release and larger inhibition zones. Drug release profiles were best described by Weibull and first-order models, showing an initial burst followed by sustained release. These findings indicate that CAP spraying enables solvent-free fabrication of antibiotic-loaded PVA coatings with tunable release, and optimizing spraying speed improves coating mass, drug delivery, and antibacterial performance.

## 1. Introduction

Post-operative infections associated with metal implants remain a major challenge in clinical practice, often leading to prolonged hospitalization, increased treatment costs, and, in severe cases, implant failure or removal [1]. To mitigate these risks, localized antibiotic delivery directly from implant surfaces has attracted significant attention as a strategy to provide high local drug concentrations while minimizing systemic side effects [2].

**Figure 1:**
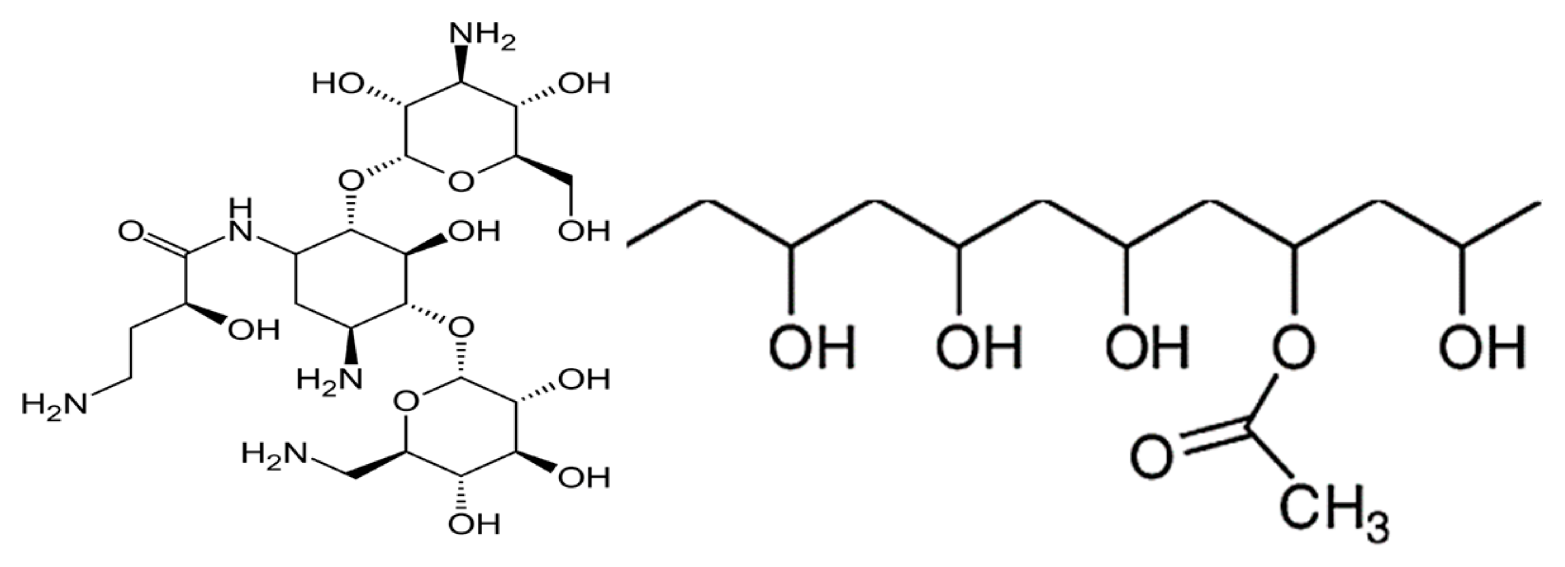
Chemical structures of amikacin (**left**) and polyvinyl alcohol (PVA, **right**).

Biodegradable polymers, particularly polyvinyl alcohol (PVA) (Figure 1, right), have gained interest due to their excellent biocompatibility, hydrophilicity, and ability to sustain and control drug release [3,4]. However, traditional coating techniques such as dip-coating or solvent casting present significant limitations [5]. Dip-coating, for instance, frequently results in non-uniform film thickness and poor adhesion, increasing the risk of delamination. Furthermore, the elevated temperatures often required during post-deposition processing can introduce surface defects and compromise coating quality [6].

Cold atmospheric pressure plasma (CAP) deposition offers a promising solvent-free alternative that allows precise control over surface properties, thin-film formation, and coating adhesion, while providing a rapid, clean, and environmentally friendly process [7]. CAP has been widely used to tailor surface energy, improve polymer-substrate adhesion, and control thin-film morphology [8]. Nevertheless, despite extensive use of cold atmospheric plasma for surface modification, its application in plasma-assisted deposition of biodegradable, antibiotic-loaded polymer coatings with controlled drug release has been comparatively underexplored. Moreover, the influence of CAP parameters, particularly nozzle distance and plasma flow speed, on coating structure, drug-release kinetics, and antibacterial efficacy is not yet fully understood, especially in the context of plasma-assisted deposition of biodegradable, drug-loaded polymer coatings.

The present study aims to optimize CAP deposition of PVA–amikacin (Figure 1, left) coatings on stainless steel substrates to achieve sustained antibiotic release and enhanced antibacterial activity. A 3 × 3 factorial design was employed to systematically investigate the effects of plasma nozzle distance and spraying speed on coating surface morphology, drug release kinetics, and antimicrobial performance. We hypothesize that specific CAP parameter combinations will produce coatings with desirable surface roughness and porosity, leading to more effective and prolonged antibiotic release. These insights could inform the development of next-generation biodegradable antimicrobial coatings for medical implants.

## 2. Materials and Methods

### 2.1. Materials

Substrate: Stainless steel foil (AISI 304, Hasberg Schneider GmbH, Rudolf-Stratz-Straße 183233 Bernau am Chiemsee, Germany) with thicknesses of 100 µm or 200 µm (depending on availability).

Poly(vinyl alcohol) 99% (Parteck^®^ SRP 80, EMPROVE^®^ ESSENTIAL, Cat. No. 141439) was obtained from Merck KGaA (Darmstadt, Germany).

Amikacin (≥99% purity) was obtained from Sintez Pharmaceutical Company (Kurgan, Russia).

### 2.2. Methods

#### 2.2.1. Sample Preparation

The foil was cut into 15 × 15 cm pieces and pre-cleaned with ethanol. Before plasma spraying, the substrate was cleaned with pure argon plasma at a distance of 25 mm and a nozzle speed of 20 cm/s. The drug-polymer mixture was deposited onto the foil using cold atmospheric plasma (CAP).

#### 2.2.2. Plasma Coating Procedure

Plasma spraying was conducted using a Cold Atmospheric Plasma (CAP) system (Plasma Coat PCU3D) with a plasma current of 100 A and voltage of 26 V. Argon was used as the carrier gas at a flow rate of 20 L/min, with the particle feed rate set to maximum. Before coating, stainless-steel substrates were pre-treated with pure argon plasma at a fixed distance of 25 mm and nozzle speed of 20 cm/s.

The coating was applied under varying nozzle speeds (10, 15, and 20 cm/s) and spraying distances (15, 20, and 25 mm), creating nine unique combinations (see Table 1). Each condition was replicated with a specific substrate thickness (100 μm or 200 μm).

#### 2.2.3. Sample Storage

All coated and plasma-treated samples were stored in zipper storage bags at ambient temperature to prevent microbial contamination and moisture exposure prior to characterization and testing.

### 2.3. Characterization

#### 2.3.1. Surface Roughness

**Figure 2:**
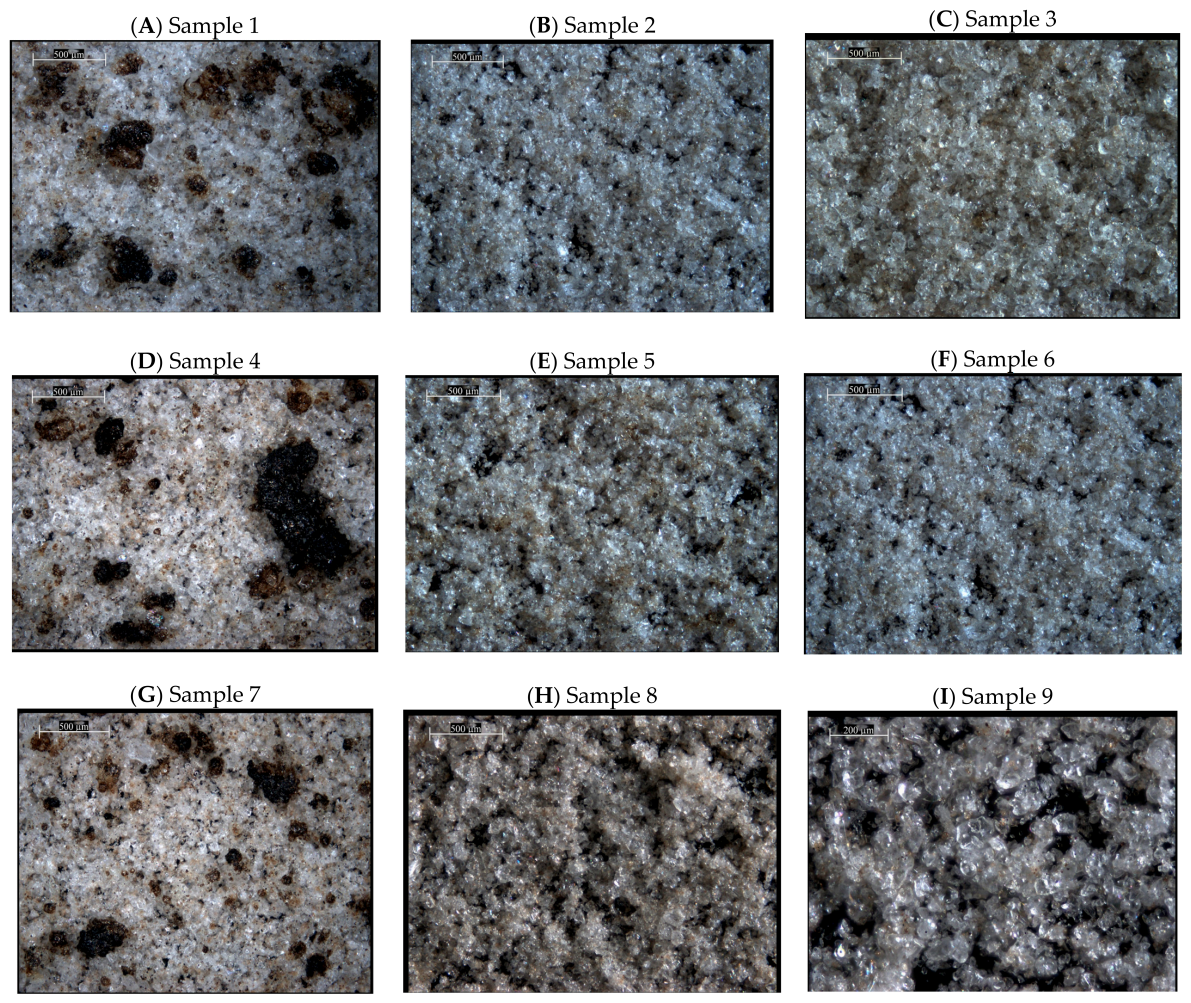
Optical microscopy images (×5) of PVA–amikacin coatings deposited on stainless steel by cold atmospheric pressure plasma spraying at different nozzle distances and spraying speeds. (**A**) Sample 1: 15 mm, 10 cm/s. (**B**) Sample 2: 20 mm, 10 cm/s. (**C**) Sample 3: 25 mm, 10 cm/s. (**D**) Sample 4: 15 mm, 15 cm/s. (**E**) Sample 5: 20 mm, 15 cm/s. (**F**) Sample 6: 25 mm, 15 cm/s. (**G**) Sample 7: 15 mm, 20 cm/s. (**H**) Sample 8: 20 mm, 20 cm/s. (**I**) Sample 9: 25 mm, 20 cm/s. Lower nozzle distances and speeds produced rougher, more porous coatings, whereas higher speeds and longer distances yielded smoother, thinner films.

Surface morphology and topography were analyzed using an optical microscope (DM 6000M, Leica Microsystems, Wetzlar, Germany) operated in dark-field mode. To enhance depth of focus and visualize surface features with varying height, a focus stacking technique was employed. For each sample, a series of 20–25 images was captured at different focal planes depending on surface roughness (Figure 2). These images were processed using Helicon Focus 5.3 Pro software to generate a single high-clarity 2D image.

#### 2.3.2. Antimicrobial Activity

**Figure 3:**
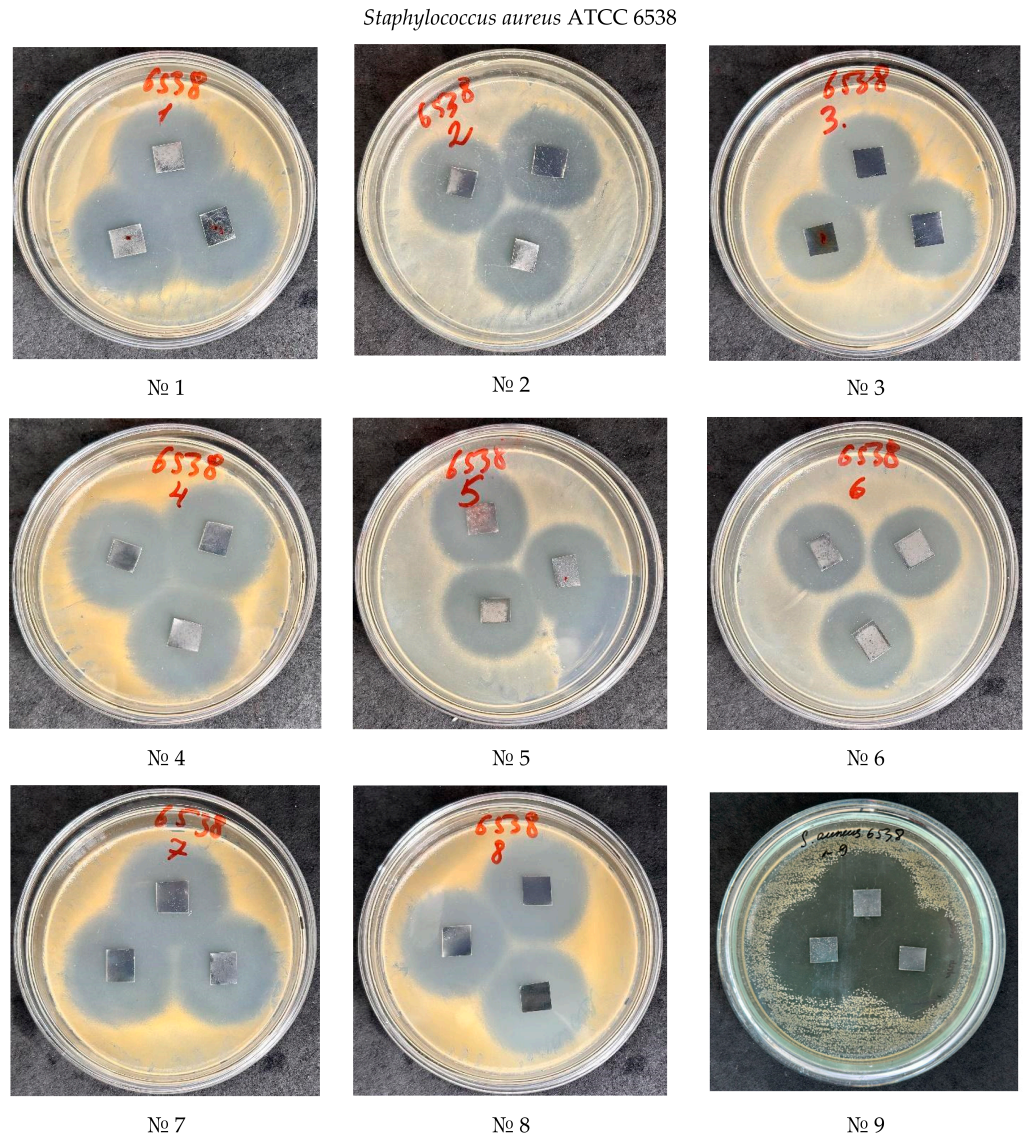
Representative agar diffusion assay against *Staphylococcus aureus* ATCC 6538 for PVA–amikacin coatings prepared under different cold atmospheric pressure plasma (CAP) conditions. Plates № 1–№ 9 correspond to Samples 1–9 in Table 1: (1) 15 mm, 10 cm/s; (2) 20 mm, 10 cm/s; (3) 25 mm, 10 cm/s; (4) 15 mm, 15 cm/s; (5) 20 mm, 15 cm/s; (6) 25 mm, 15 cm/s; (7) 15 mm, 20 cm/s; (8) 20 mm, 20 cm/s; (9) 25 mm, 20 cm/s. Each Petri dish shows three replicate coated squares per condition.

**Figure 4:**
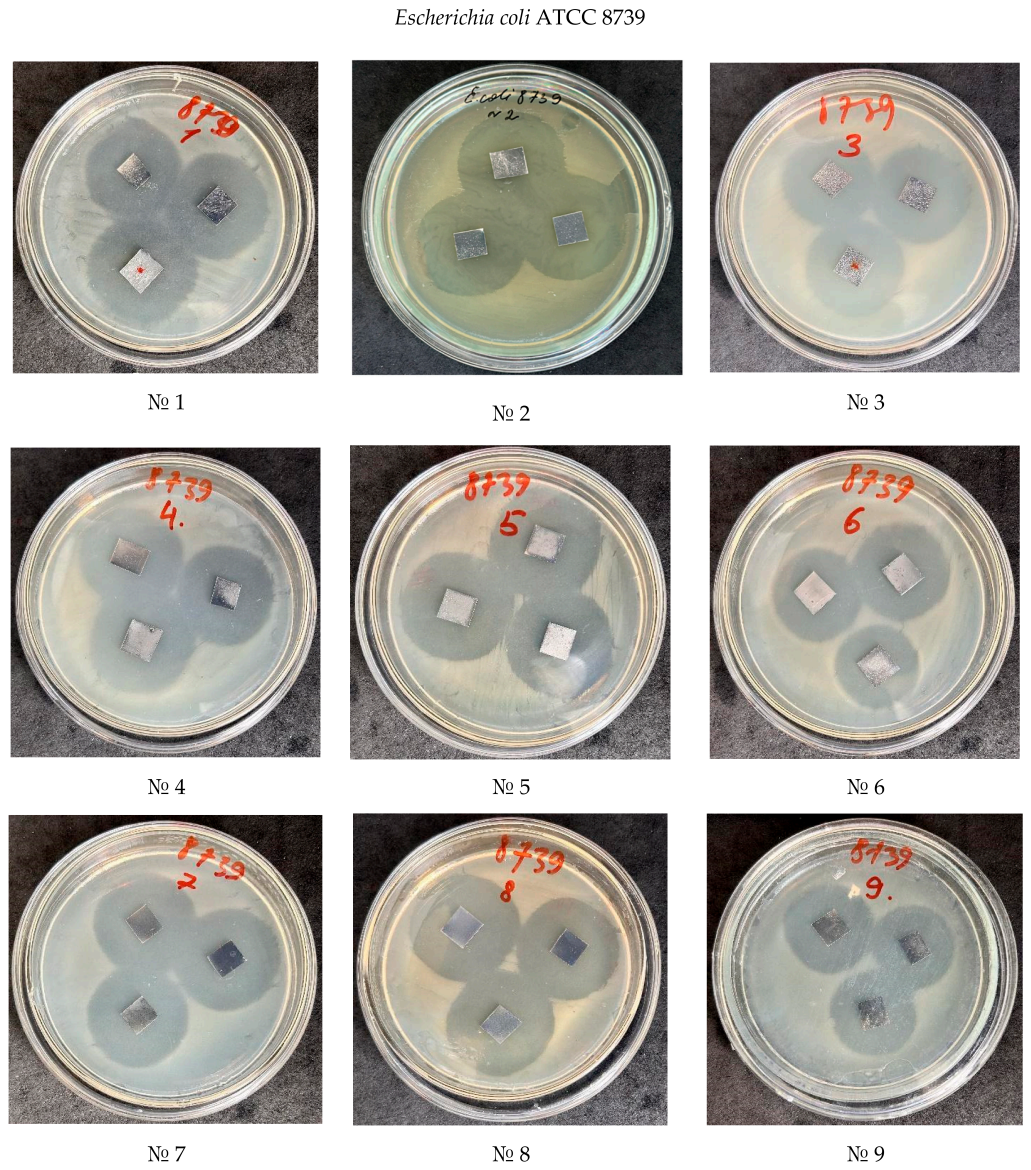
Representative agar diffusion assay against *Escherichia coli* ATCC 8739 for PVA–amikacin coatings prepared under different CAP conditions. Film numbers № 1–№ 9 correspond to the same coating preparations as in Figure 3 and Table 1: (1) 15 mm, 10 cm/s; (2) 20 mm, 10 cm/s; (3) 25 mm, 10 cm/s; (4) 15 mm, 15 cm/s; (5) 20 mm, 15 cm/s; (6) 25 mm, 15 cm/s; (7) 15 mm, 20 cm/s; (8) 20 mm, 20 cm/s; (9) 25 mm, 20 cm/s.

Antibiotic efficiency was evaluated using the Kirby–Bauer disc diffusion method. Samples were placed on agar plates inoculated with either Gram-positive (ATCC 6538) or Gram-negative (*Escherichia coli* ATCC 8739) bacteria (Figure 3 and Figure 4). Plates were incubated at 37 °C for 24–48 h, after which zones of inhibition were measured in millimeters [9].

#### 2.3.3. Drug Release Profile

In vitro release profiles of amikacin were determined by immersing coated samples in the HPLC mobile phase. Antibiotic concentrations in the samples were quantified using HPLC-HRMS as described in Section 2.3.4, and cumulative release was plotted as drug release percentage versus time.

The release percentage (%R) was calculated using the equation:

$$\%R\;=\;\frac{Mt}{M\infty }\times \;100$$

where Mt is the mass of amikacin released at time t, and M∞ is the total amount of amikacin incorporated in the coating (determined from complete extraction at the end of the experiment).

#### 2.3.4. HPLC-HRMS Analysis of Amikacin

Quantitative analysis of amikacin in the release medium was performed using a Vanquish UHPLC system coupled to a Q Exactive HF Orbitrap high-resolution mass spectrometer (Thermo Fisher Scientific, Hanna-Kunath-Str. 11, 28199 Bremen, Germany). Separation was achieved using a Zorbax SB-C8 column (2.1 × 100 mm, 1.8 µm; Agilent, USA). The mobile phases consisted of (A) 0.1% formic acid in Milli-Q water and (B) HPLC-grade acetonitrile.

The gradient program was as follows:
**Time****Mobile Phase Composition (% B)**0–5.0 min100% B5.0–7.5 minlinear gradient to 0% B7.5–9.0 minheld at 0% B9.0–9.001 minramp to 100% B9.001–11.0 minre-equilibration at 100% B

The flow rate was set at 0.45 mL/min with a 2.0 μL injection volume. The column was maintained at 25 °C and the autosampler at 12 °C. Mass spectrometry was performed in positive electrospray ionization (ESI) mode with the following parameters: sheath gas 50, sweep gas 1, auxiliary gas 30 (arbitrary units); spray voltage 4.0 kV; capillary temperature 280 °C; in-source CID energy 80 eV.

Data acquisition was done in two modes. First, full scan mode was used over the mass range of 50–750 m/z with an AGC target of 3 × 10^6^ and a resolution of 15,000. Second, Parallel Reaction Monitoring was conducted for amikacin at m/z 586.2930, with a normalized collision energy of 30, a resolution of 30,000, an AGC target of 5 × 10^5^, and an isolation window of 4 m/z. Quantification was based on a five-point calibration curve (0.1–10 µg/mL), with R^2^ > 0.999.

### 2.4. Data Analysis

The dataset comprised 68 observations, each representing drug concentration at defined speed (10, 15, or 20 cm/s) and distance (15, 20, or 25 mm) combinations. Each speed group included 22–24 samples distributed across distance levels.

To describe the release kinetics, experimental data were fitted to multiple mathematical models commonly applied to drug release studies [10,11]: zero-order (Mt = k0t), first-order (Mt = 1−exp(−k1t)), Higuchi $\left(Mt\;=\;kH\sqrt{t}\right)$, Hixson–Crowell $\left(Mt\;=\;1-(1-kHCt{)}^{3}\right)$, Korsmeyer–Peppas $\left(Mt/M\infty =kHPt^{n}\right)$, Weibull $\left(Mt/M\infty \;=\;1-exp[-(t/\tau {)}^{\beta }]\right)$, linear, logarithmic, polynomial (second-order), and exponential rise-to-maximum models.

For each plate, all models were tested and compared using goodness-of-fit criteria (coefficient of determination $R^{2}$, residual sum of squares [RSS], and Akaike Information Criterion [AIC]). The model with the highest R^2^ and lowest AIC was selected as the best-fit description of the release profile [12,13] (see Appendix B).

Statistical analyses were performed in Google Colaboratory using Python 3.12.13. Data processing and organization were conducted using pandas (v2.2.2). Two-way ANOVA models were constructed using statsmodels (v0.14.6) to evaluate the effects of nozzle speed, spraying distance, and their interaction on drug release. Post hoc pairwise comparisons between speed groups were performed using Tukey’s Honestly Significant Difference (HSD) test where appropriate. Because assumption checks indicated violations of normality and homogeneity of variance, nonparametric validation was performed using the Kruskal–Wallis test followed by Mann–Whitney U tests with Bonferroni correction. Statistical analyses were conducted using SciPy (v1.16.3) and statsmodels (v0.14.6). Data visualization was performed using matplotlib (v3.10.0) and seaborn (v0.13.2). Statistical significance was defined as *p* < 0.05.

### 2.5. Data and Material Availability

All data, materials, and protocols used in this study are available from the corresponding author upon request. There are no restrictions on data or material availability.

## 3. Results

Stainless steel foil (AISI 304) was used as the substrate material. Each foil was pre-coated with a PVA release layer and cut into uniform 2 cm × 2 cm squares to standardize surface area across all samples.

Polymers and Antibiotics: PVA (Sigma-Aldrich Chemie GmbH, Eschenstr. 5, 82024 Taufkirchen, Germany, Cat. No. 141439) was employed as a sacrificial release layer in film preparation. Amikacin was incorporated into the PVA matrix at a 1:2 ratio to provide antimicrobial function.

**Figure 5:**
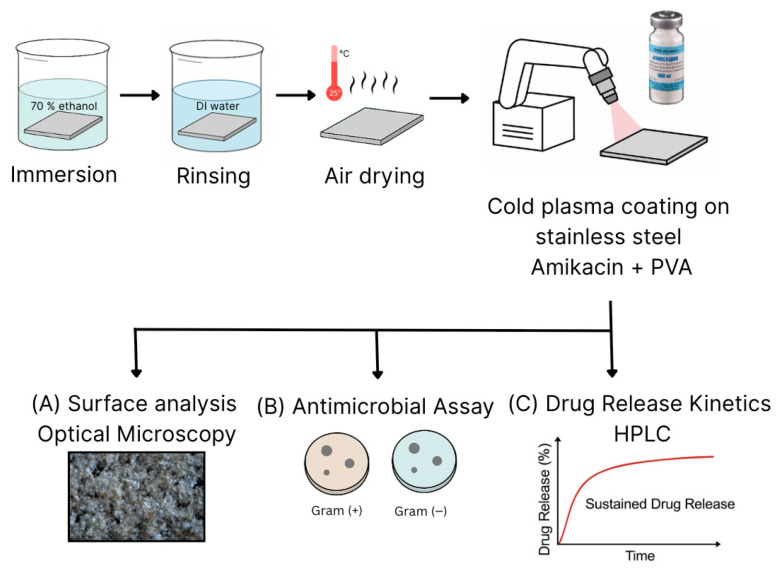
Schematic overview of the experimental workflow.

Schematic overview of the experimental workflow is shown in Figure 5.

**Figure 6:**
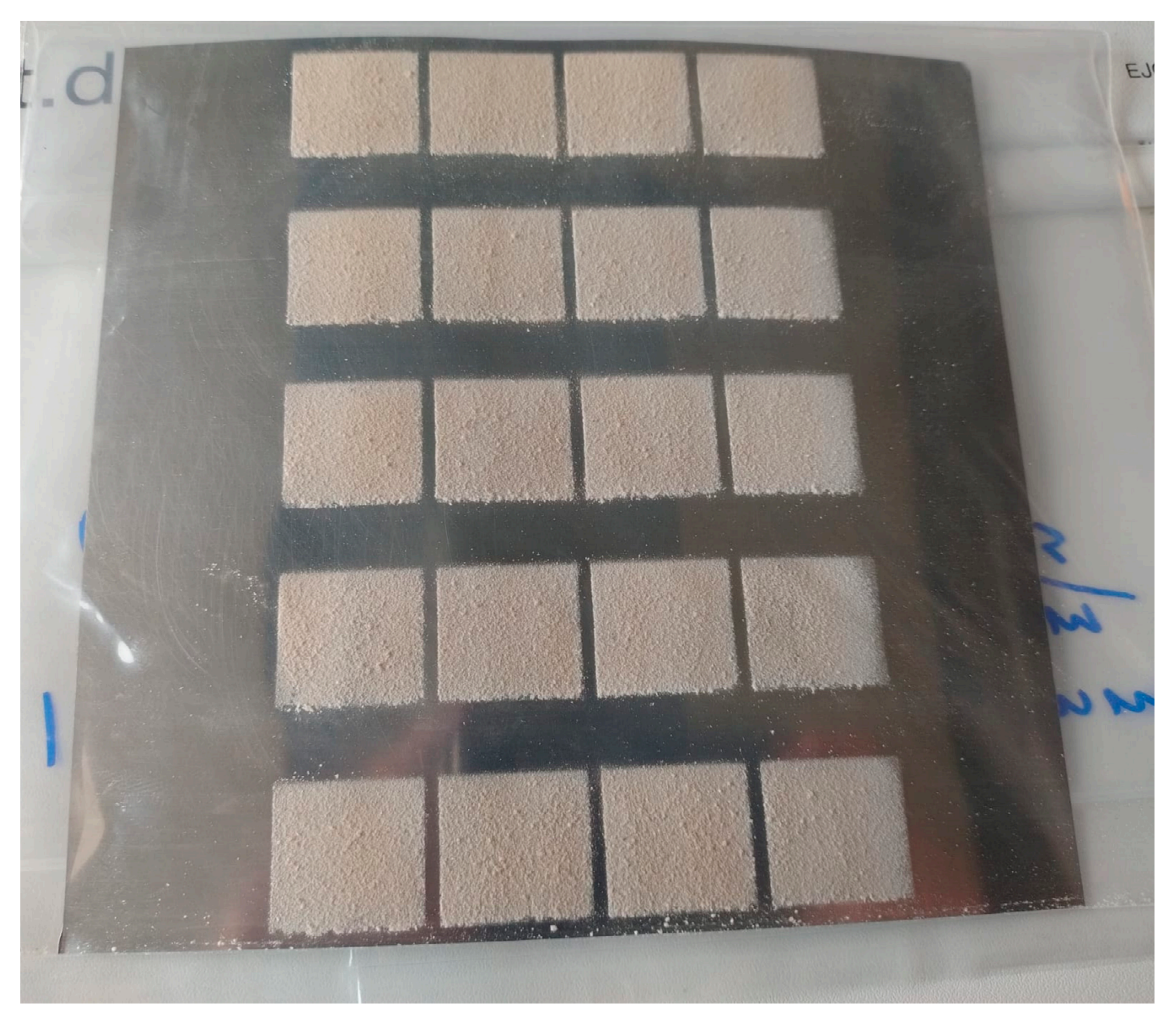
Stainless steel foil and applied PVA + amikacin.

Resulted stainless steel foil with applied PVA+amikacin is shown in Figure 6.

### 3.1. Coating Mass and Plasma Parameters

Plasma spraying produced coatings with masses ranging from 150 to 490 mg, depending on nozzle speed and spraying distance (Table 1). Lower spraying speeds (10 cm/s) at shorter distances (15–20 mm) resulted in the highest coating mass (490 mg), while higher speeds (20 cm/s) and longer distances (25 mm) yielded the lowest mass (150–200 mg). Substrate thickness (100 µm vs. 200 µm) had no apparent influence on mass deposition.

Coating mass values represent single measurements per condition; however, reproducibility was assessed indirectly through the consistency of drug release profiles and statistical analysis across multiple observations (*n* = 68).

Although a general decrease in coating mass with increasing distance is observed, minor deviations (e.g., between Samples 8 and 9) indicate that distance has a weaker and non-monotonic influence compared to nozzle speed, consistent with the non-significant ANOVA result.

### 3.2. Surface Morphology and Roughness

**Figure 7:**
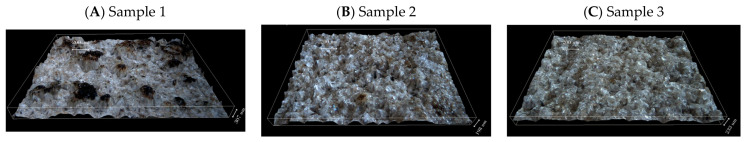

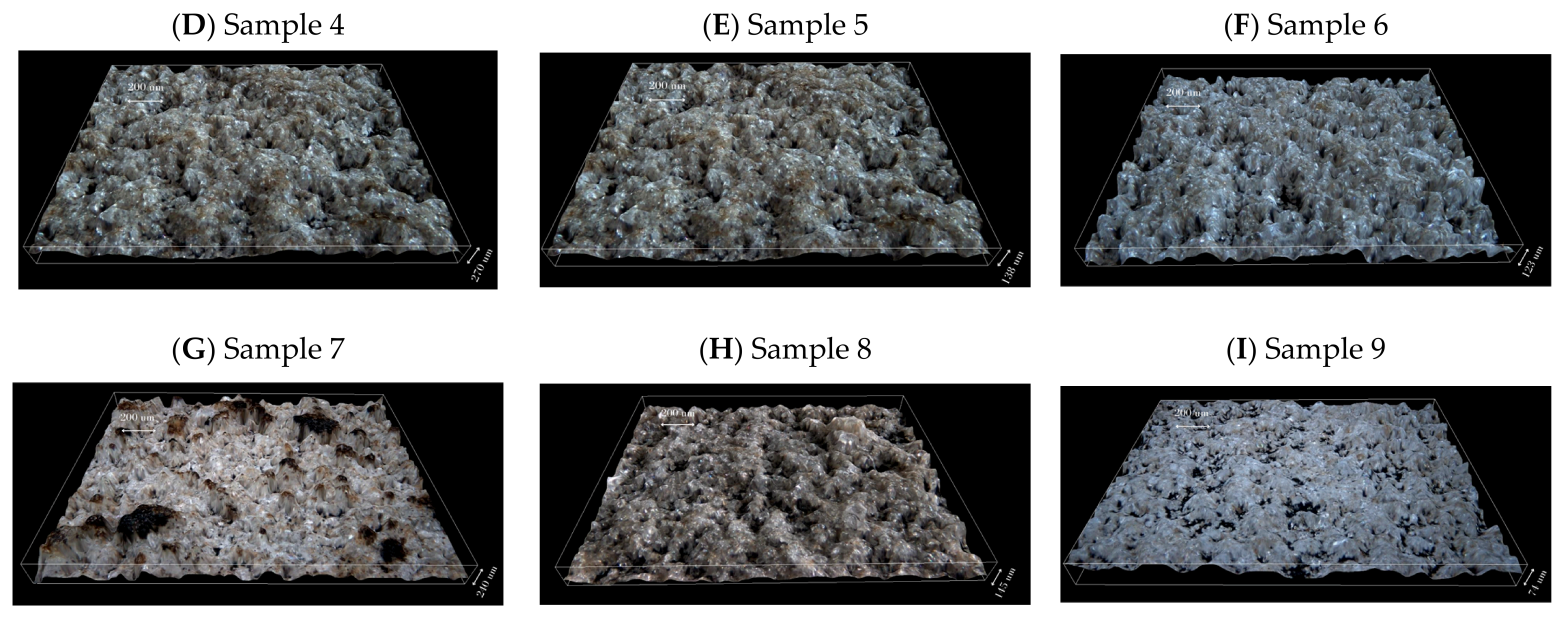
3D surface renderings from optical microscopy (×5) of the same PVA–amikacin coatings as in Figure 2, illustrating the effect of plasma parameters on surface roughness and porosity. (**A**–**I**) Coating conditions correspond to those in Figure 2.

Optical microscopy revealed distinct morphological differences among coatings (Figure 2). At short nozzle distances (15 mm) and low speeds (10 cm/s), surfaces appeared rougher and more porous, while longer distances (25 mm) produced thinner, smoother films. Depth profiles confirmed that rougher surfaces corresponded to increased porosity and higher surface area (Figure 7).

### 3.3. Drug Release Profiles

Drug release from the PVA–amikacin coatings was well described by the Weibull model for all nine plates (Table 2). The fits were excellent, with coefficients of determination in the range $R^{2}$ values ranging from 0.902 to 0.996 and AIC values between −29.8 and −54.6. Plates 1 and 2 showed the best agreement with the model (both $R^{2}$ = 0.996; AIC = −54.0 and −54.6, RSS = 0.00565 and 0.0053, respectively). Plates 3, 5, 7, and 8 also presented very good fits ($R^{2}$ = 0.982–0.993, AIC = −35.9 to −45.8), while Plates 4, 6, and 9 still showed acceptable fits despite slightly lower $R^{2}$ values and higher RSS (0.0288–0.1167).

For all coatings, the characteristic time parameter of the Weibull function ${\left(t/\tau \right)}^{\beta }$ fell within τ = 63.7–264.5 min, indicating slow, prolonged release. The shape parameter β was >1 for eight of the nine plates (1.03–5.00), consistent with predominantly sigmoidal release kinetics and suggesting complex, diffusion-dominated transport rather than simple first-order release. Plates 1, 6, and 8, in particular, displayed high β values (2.35, 3.99, and 5.00), corresponding to a pronounced acceleration phase in the middle of the release profile. Plate 9 was the only sample with β < 1 (β = 0.688), indicative of a more pronounced initial burst followed by a progressively slower release phase.

**Figure 8:**
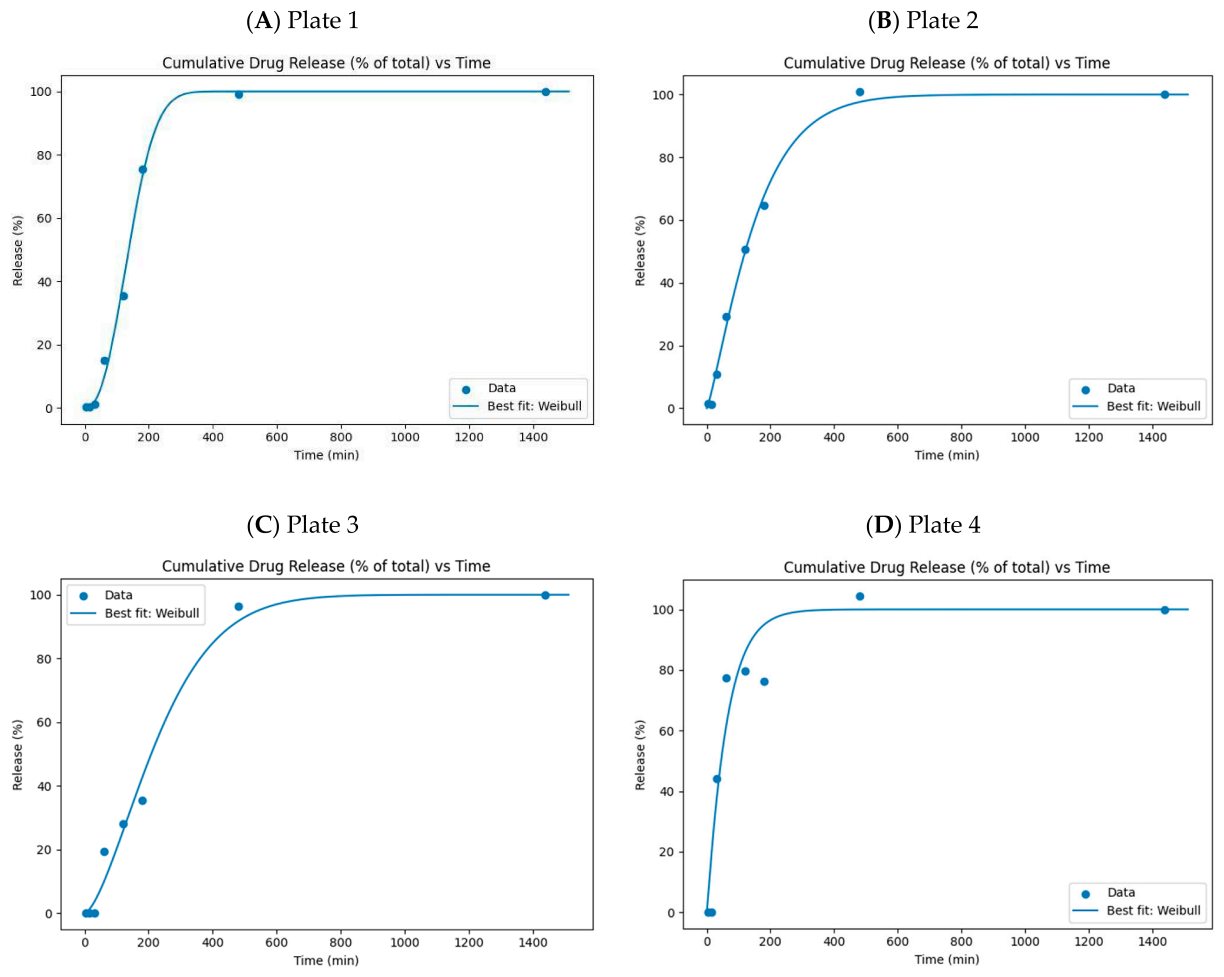

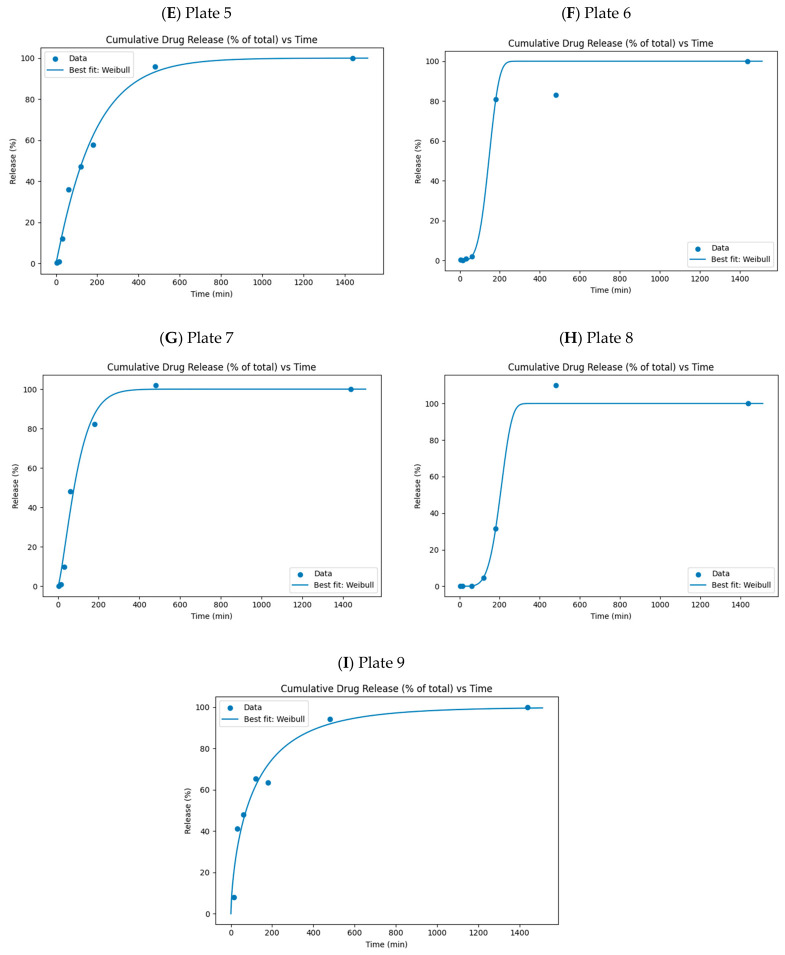
Experimental drug release profiles with best-fit kinetic model curves for Plates 1–9.

Experimentally, the coatings released approximately 45–80% of their loaded amikacin over the studied period (Figure 8), demonstrating sustained release behavior. All release profiles were biphasic, with an initial burst phase followed by a slower, diffusion-controlled release consistent with the Weibull fits.

### 3.4. Antimicrobial Activity

Kirby–Bauer assays confirmed functional antibacterial performance (Table 3, Figure 3 and Figure 4). Samples with higher cumulative release (e.g., Plates 1–2) generated larger inhibition zones (37–40 mm for *S. aureus*, ~37 mm for *E. coli*). In contrast, Plates 6 and 9, with lower release (~30–45%), produced smaller inhibition zones (28–32 mm).

### 3.5. Correlation of Drug Release with Antimicrobial Activity

**Figure 9:**
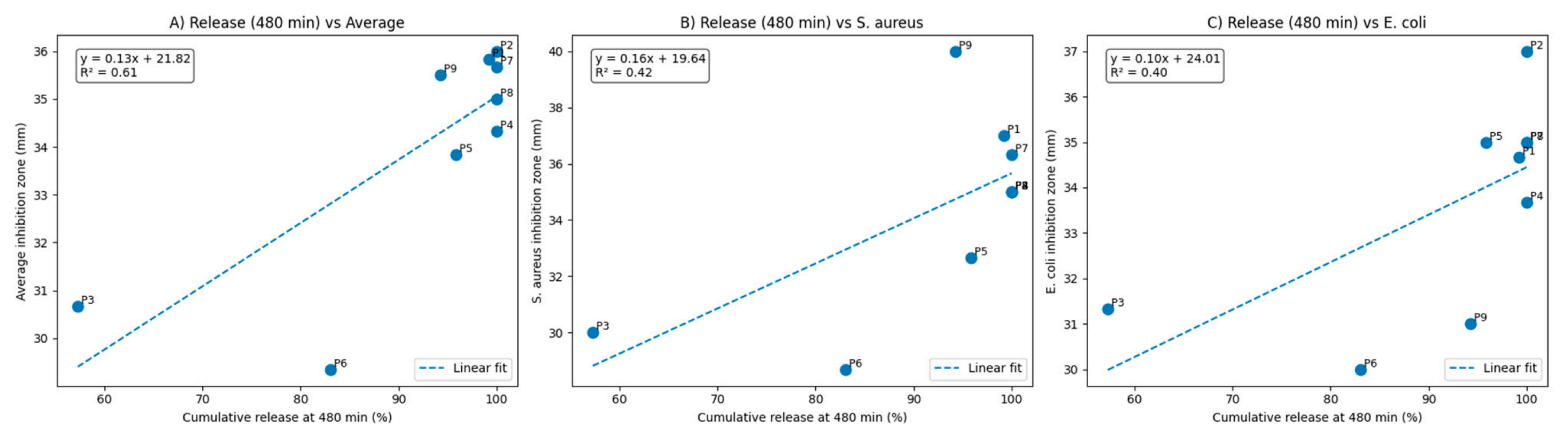
Correlation between release percentage and inhibition zone size for Plates 1–9.

To directly compare release kinetics with antibacterial activity, cumulative release percentages at 60 min and 480 min were paired with inhibition zone diameters against *S. aureus* and *E. coli* (Table 4). Average inhibition zones were calculated as the means of the two bacterial strains. A scatter plot (Figure 9) was generated to visualize the relationship between release percentage and inhibition zone size.

### 3.6. Statistical Analysis

**Figure 10:**
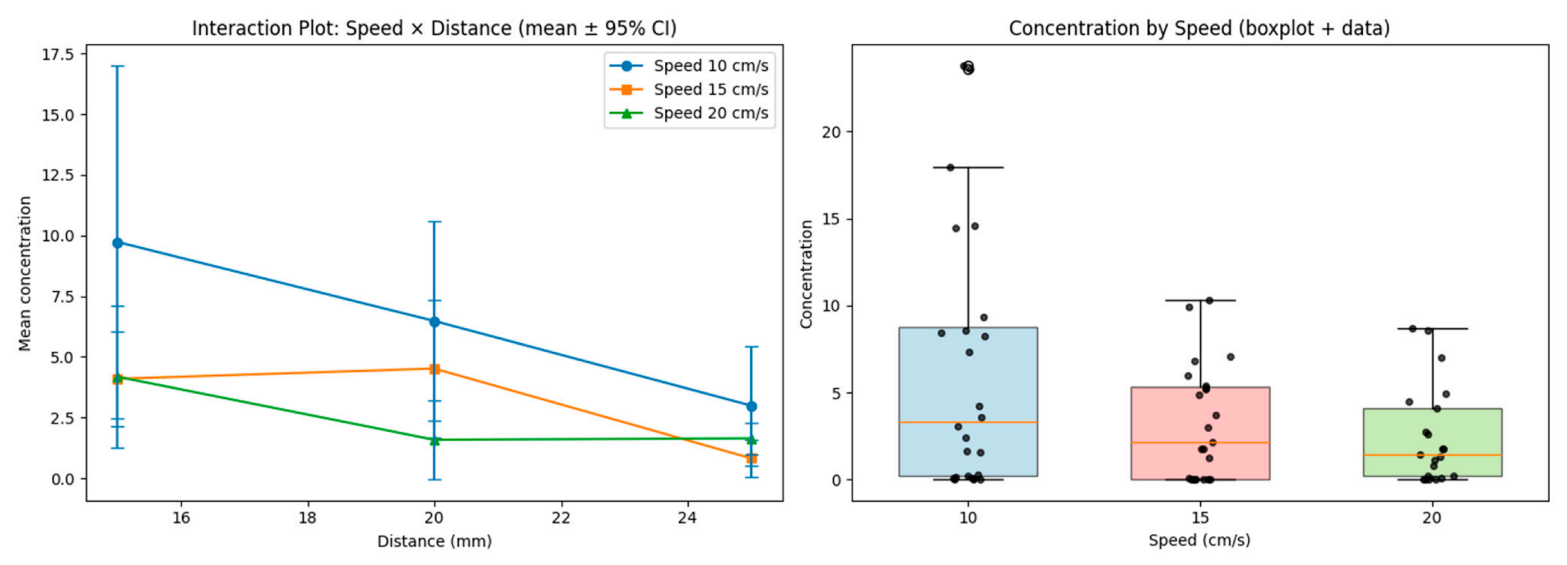
Effect of plasma parameters on drug release. (**Left**) Interaction plot showing mean concentration (±95% CI) across nozzle distance (15, 20, 25 mm) for each speed (10, 15, 20 cm/s). Lines are approximately parallel, consistent with a non-significant speed × distance interaction [F(4,63) = 0.64, *p* = 0.638]. Error bars in the panel denote 95% confidence intervals. (**Right**) Boxplots of concentration grouped by speed with individual observations overlaid. Distributions at 20 cm/s (green) are shifted lower than at 10 (blue) and 15 cm/s (red), supporting the significant main effect of speed (F(2,63) = 5.60, *p* = 0.0058) and Tukey contrasts (20 cm/s < 10 cm/s, *p* = 0.0053; 20 cm/s < 15 cm/s, *p* = 0.0465; 10 cm/s vs. 15 cm/s, n.s.).

Two-way ANOVA revealed a significant main effect of speed on drug release (F(2,63) = 5.60, *p* = 0.0058), whereas distance (F(2,63) = 1.41, *p* = 0.252) and the speed × distance interaction (F(4,63) = 0.64, *p* = 0.638) were not significant (Figure 10 (left); Table 5). Post hoc Tukey analysis showed that drug release at 20 cm/s was significantly lower than at 10 cm/s (*p* = 0.0053) and 15 cm/s (*p* = 0.0465), while no difference was observed between 10 and 15 cm/s (Figure 10 (right); Table A1, see Appendix A.2).

Assumption checks indicated violations of normality (Shapiro–Wilk, *p* < 0.05) and homogeneity of variance (Levene’s test, *p* = 0.005). To confirm robustness, nonparametric testing was performed. The Kruskal–Wallis test identified significant group differences (H = 17.78, *p* < 0.001), and Mann–Whitney U tests with Bonferroni correction confirmed that 20 cm/s differed significantly from both 10 cm/s (*p* < 0.001) and 15 cm/s (*p* = 0.0017), while 10 vs. 15 cm/s remained non-significant (Table A2, see Appendix A.2).

Together, these results demonstrate that nozzle speed is the dominant factor governing drug release, while spraying distance has no independent or interactive effect.

## 4. Discussion

### 4.1. Coating Mass and Plasma Parameters

Our results show that plasma spraying can produce coatings with masses ranging from 150 to 490 mg, depending on the nozzle speed and spraying distance (Table 1). The greatest deposition was achieved with lower spraying speeds (10 cm/s) at short distances (15–20 mm), while substantially lower coating mass was produced with higher speeds (20 cm/s) and longer distances (25 mm). This relationship can be attributed to particle residence time: at lower nozzle speeds, plasma-entrained particles have more time to settle and adhere to the substrate, whereas higher speeds reduce contact time and deposition efficiency. Similar trends have been reported in the plasma spraying of polymers and ceramics, where nozzle speed and stand-off distance are critical factors governing film thickness and uniformity. Interestingly, substrate thickness (100 µm vs. 200 µm) had no effect on deposition, indicating that heat dissipation and stability were sufficient in both cases. These findings suggest that nozzle dynamics, rather than substrate characteristics, are the primary determinants of coating yield in CAP [14].

Unlike solvent-cast PVA films, which typically form homogeneous and relatively dense matrices, CAP-deposited coatings exhibit heterogeneous, plasma-generated architectures characterized by variable porosity and surface roughness. Optical microscopy confirmed that plasma parameters directly control these features, with low nozzle speeds producing rough, porous coatings and high speeds yielding thinner, smoother films.

This structural heterogeneity is reflected in the release kinetics. The predominance of Weibull behavior with β > 1 suggests complex transport mechanisms involving combined diffusion and structural effects, rather than the simple Fickian diffusion commonly reported for solvent-cast systems.

Therefore, CAP deposition introduces an additional level of control over drug release by tailoring microstructure through processing conditions, which is not readily achievable with conventional solvent-based methods.

### 4.2. Surface Morphology and Roughness

Microscopic analysis revealed distinct morphological differences (Figure 2 and Figure 7). At short nozzle distances and low speeds, the surfaces appear rougher and more porous, while long distances yield smoother films with reduced porosity. Optical microscopy depth analysis confirmed that the increased roughness corresponds to a higher surface area, a property known to influence drug loading capacity and release kinetics. Previous works on plasma-modified polymer coatings support this observation, as increased porosity enhances fluid penetration and accelerates drug diffusion [15]. In the context of antimicrobial implants, rougher surfaces may also improve tissue integration [16], though excessively porous films could compromise coating integrity. Thus, controlling the plasma parameters offers a route to balancing adhesion, porosity, and release performance.

### 4.3. Drug Release Profiles

Drug release studies demonstrated sustained biphasic release from all coatings (Table 2, Figure 8). Each profile showed an initial burst phase followed by a slower, diffusion-controlled stage, consistent with the typical behavior of PVA matrices, where rapid release originates from surface-associated drug while prolonged release is governed by diffusion through the hydrated polymer bulk [17,18,19]. Experimentally, the coatings released approximately 45–80% of their loaded amikacin over the studied period, indicating sustained but partial drug depletion from the films. Drug release from the PVA–amikacin coatings was well described by the Weibull model for all nine plates (Table 2). The fits were excellent, with coefficients of determination $R^{2}$ ranging from 0.902 to 0.996 and AIC values between −29.8 and −54.6.

For some samples (e.g., Plate 4), the Weibull model provided a comparatively lower fit (R^2^ ≈ 0.90), suggesting that more complex or multi-phase release mechanisms may be present; however, a unified modeling approach was retained to allow consistent comparison across conditions.

The kinetic parameters also reflected the influence of the plasma spraying conditions. At a fixed nozzle speed of 10 cm/s (Plates 1–3), increasing the spraying distance from 15 to 25 mm shifted the profiles toward slower, more extended release: t_50_ values were 136.98, 120.69 and 208.15 min, respectively, with Plate 3 (25 mm) showing the largest scale parameter τ (264.5 min) and the slowest release (t_80_ = 361 min). At a fixed distance of 15 mm (Plates 1, 4, 7), increasing nozzle speed from 10 to 20 cm/s reduced coating mass (490 → 330 → 240 mg) and accelerated early release (t_10_ = 61.45 → 7.47 → 19.38 min), while altering the shape parameter (β = 2.35 → 1.05 → 1.34), consistent with more heterogeneous coating microstructures. The slowest, most sustained release was observed for Plate 3 (25 mm, 10 cm/s), whereas Plate 4 (15 mm, 15 cm/s) released 80% of its load in ~100 min, representing the fastest profile. Overall, coating mass broadly correlated with total release time, but deviations such as Plate 8 (low mass, 150 mg, yet very high β = 5) suggest that microstructural features generated by specific plasma parameter combinations also play a critical role in governing drug transport. These observations highlight that CAP spraying offers a powerful handle to tune antibiotic release kinetics, but also that careful optimization of both distance and speed is required to avoid excessive burst release while maintaining a therapeutically relevant, sustained delivery window. Comparable sustained or controlled-release behaviour has been reported for PVA-based films and hydrogels prepared by conventional solvent-based methods, including simple PVA matrices and PVA blends with chitosan, gelatin, hyaluronic acid, or other polysaccharides for ocular and wound-healing applications [20,21,22,23,24,25,26,27].

### 4.4. Antimicrobial Activity

Kirby–Bauer assays confirmed the antibacterial functionality of the PVA–amikacin films (Table 3; Figure 3 and Figure 4). Coatings with a higher release (e.g., Plates 1–2) yielded larger inhibition zones (~35–40 mm), while those with a lower release (~30–45%) produced smaller zones (28–32 mm). This direct relationship supports the biological relevance of the release profiles. Notably, both *S. aureus* and *E. coli* were effectively inhibited, demonstrating broad-spectrum efficacy. Previous studies have highlighted amikacin’s suitability for implant-associated infections due to its stability and activity against resistant strains [28]. Comparable local delivery approaches include amikacin-loaded porous PLA/PVA matrices, which generate inhibition zones of ~7–9.5 mm against *S. aureus* and *E. coli* while providing multi-day release, and amikacin-coated 3D-printed stainless-steel implants, which produce inhibition areas of 0.9–3.0 cm^2^ (≈10–20 mm equivalent diameter) and maintain antibacterial activity for up to 7 days [29,30]. Encapsulation of amikacin into degradable PLA-based microparticles has also been shown to support prolonged release at concentrations exceeding MIC values for common pathogens [31]. In this context, our CAP-deposited PVA coatings achieve relatively large inhibition zones after 24 h, indicating at least comparable short-term antibacterial efficiency, although direct comparison is limited by differences in sample geometry, drug loading, and assay conditions [29,30]. Our results further suggest that plasma-deposited PVA coatings can serve as an efficient local delivery platform, potentially reducing the need for systemic antibiotic administration and minimizing associated side effects.

Although the chemically defined medium was optimized for the present system, its applicability to other thraustochytrid species remains to be evaluated, and future studies should investigate its performance across diverse strains.

### 4.5. Correlation of Drug Release with Antimicrobial Activity

Correlation analysis revealed moderate to strong positive associations between release percentage (at 60 and 480 min) and inhibition zone diameters (Figure 9; Table 4). The strongest correlations were observed for cumulative release at 480 min, which aligns with antibacterial outcomes measured at 24 h. This suggests that early release kinetics critically shape antimicrobial efficacy in vitro [32]. The weaker correlations at 60 min reflect the fact that, while measurable, initial burst release is not always sufficient to fully explain inhibition zone sizes, which integrate drug exposure over time [33,34]. These findings highlight the importance of sustained release for maintaining antibacterial activity, suggesting that plasma-optimized coatings capable of extended release will be the most clinically effective.

### 4.6. Statistical Analysis

Statistical evaluation confirmed that nozzle speed was the dominant factor governing drug release [35], while spraying distance had no significant main or interaction effect (Figure 10; Table 5). ANOVA identified significant differences between speeds, with 20 cm/s producing consistently lower release compared to 10 and 15 cm/s. Tukey and Mann–Whitney tests confirmed these trends, though no difference was observed between 10 and 15 cm/s. The robustness of these findings across parametric and nonparametric approaches underscores the reliability of speed as the key controllable factor. From a mechanical perspective, higher speeds reduce residence time and deposition efficiency, resulting in thinner coatings with less drug loading and reduced release. These insights provide practical guidance for optimising the plasma process, emphasizing speed control over distance adjustment [36].

### 4.7. Broader Implications and Future Directions

Collectively, our findings support the feasibility of CAP as a versatile platform for producing antibiotic-loaded biodegradable coatings with tunable properties [37]. Unlike dip-coating or solvent casting, plasma spraying eliminates the need for solvents, minimizes processing defects, and enables precise control over film morphology and drug distribution. This makes it especially attractive for clinical translation, where reproducibility and biocompatibility are critical [38]. Future research should explore (i) incorporating different antibiotics or combinations to address resistant pathogens, (ii) multilayer coatings to decouple burst and sustained release phases, (iii) in vivo evaluation of antimicrobial efficacy and biocompatibility, and (iv) scale-up strategies for coating complex implant geometries. By addressing these areas, plasma-assisted deposition could advance the development of next-generation antimicrobial implants with improved infection control and patient outcomes.

## 5. Conclusions

This study demonstrates that cold atmospheric pressure plasma (CAP) deposition is an effective solvent-free approach for fabricating polyvinyl alcohol amikacin coatings with tunable structural and functional properties. Using a 3 × 3 factorial design (nozzle speed: 10–20 cm/s; distance: 15–25 mm), coating masses in the range of 150–490 mg were obtained, confirming strong process–structure relationships.

Nozzle speed was identified as the dominant parameter governing coating mass and drug release (two-way ANOVA: F(2,63) = 5.60, *p* = 0.0058), whereas spraying distance had no statistically significant effect (*p* = 0.252). Lower speeds produced thicker, more porous coatings with higher cumulative release, while higher speeds resulted in thinner films with reduced drug loading. Drug release profiles were consistently described by the Weibull model (R^2^ = 0.902–0.996; AIC = −29.8 to −54.6), with characteristic times τ = 63.7–264.5 h and shape parameters β = 1.03–5.00, indicating complex, non-Fickian transport mechanisms.

All coatings exhibited sustained, biphasic release behavior, delivering approximately 45–80% of the incorporated amikacin over the studied period. Antibacterial assays confirmed effective activity against *Staphylococcus aureus* and *Escherichia coli*, with inhibition zones ranging from ~28 to 40 mm, correlating with cumulative drug release.

Overall, CAP deposition enables precise control of coating architecture and drug release behavior through process parameters, providing a versatile platform for localized antibiotic delivery on medical implants. Future work should focus on in vivo validation, extension to other antimicrobial agents, and scaling the process for complex implant geometries. By directly linking plasma process parameters to functional drug delivery outcomes, this work establishes CAP deposition as a fundamentally new paradigm for designing antimicrobial coatings, moving beyond passive materials toward actively engineered, performance-driven implant surfaces.

## Figures and Tables

**Table 1:** Plasma spraying parameters and corresponding coating mass on stainless steel foil substrates. Lower nozzle speed and shorter spraying distance increased coating mass deposition. Substrate Mass = 23.41 ± 8.48 g; Substrate Thickness = 144 ± 52.7 µm (mean ± SD, *n* = 9).

Number of Sample	Spraying Distance (mm)	Nozzle Speed (cm/s)	Substrate Mass (g)	Substrate Thickness(μm)	Coating Mass (mg)
1	15	10	34.48	200	490
2	20	10	34.77	200	490
3	25	10	18.03	100	370
4	15	15	34.92	200	330
5	20	15	17.64	100	300
6	25	15	17.80	100	280
7	15	20	17.79	100	240
8	20	20	17.59	100	150
9	25	20	17.68	100	200

**Table 2:** Comparison of drug release kinetic models for PVA–amikacin films.

Plate	Best-Fit Model	$R^{2}$	AIC	RSS	Equation (Fractional Release)
1	Weibull	0.996	−54.0	0.00565	$F(t)=1-exp[-(\frac{t}{160.1}{)}^{2.35}]$
2	Weibull	0.996	−54.6	0.0053	$F(t)=1-exp[-(\frac{t}{162.98}{)}^{1.22}]$
3	Weibull	0.985	−44.8	0.0018	$F(t)=1-exp[-(\frac{t}{264.497}{)}^{1.53}]$
4	Weibull	0.902	−29.8	0.1167	$F(t)=1-exp[-(\frac{t}{63.69}{)}^{1.05}]$
5	Weibull	0.985	−45.8	0.0158	$F(t)=1-exp[-(\frac{t}{184.04}{)}^{1.034}]$
6	Weibull	0.978	−34.4	0.0288	$F(t)=1-exp[-(\frac{t}{158.6}{)}^{3.997}]$
7	Weibull	0.982	−35.9	0.0233	$F(t)=1-exp[-(\frac{t}{104.4}{)}^{1.336}]$
8	Weibull	0.993	−41.8	0.0101	$F(t)=1-exp[-(\frac{t}{218.9}{)}^{5}]$
9	Weibull	0.940	−32.9	0.0359	$F(t)=1-exp[-(\frac{t}{126.084}{)}^{0.688}]$

**Table 3:** Antimicrobial activity (Kirby–Bauer method) of coated PVA–amikacin films against *Staphylococcus aureus* ATCC 6538 and *Escherichia coli* ATCC 8739. Data are means ± SD (*n* = 3).

Substance Name	Sample Number	Zone of Inhibition, mm			
		Repetition			Average
		1	2	3	
*Staphylococcus aureus* ATCC 6538	1	37.0	37.0	37.0	37.0 ± 0.00
	2	35.0	35.0	35.0	35.0 ± 0.00
	3	30.0	30.0	30.0	30.0 ± 0.00
	4	35.0	35.0	35.0	35.0 ± 0.00
	5	32.0	33.0	33.0	32.6 ± 0.58
	6	28.0	28.0	30.0	28.6 ± 1.15
	7	35.0	37.0	37.0	36.3 ± 1.15
	8	35.0	35.0	35.0	35.0 ± 0.00
	9	40.0	40.0	40.0	40.0 ± 0.00
*Escherichia coli* ATCC 8739	1	34.0	35.0	35.0	34.7 ± 0.58
	2	38.0	38.0	38.0	38.0 ± 0.00
	3	30.0	32.0	32.0	31.3 ± 1.15
	4	33.0	33.0	35.0	33.7 ± 1.15
	5	35.0	35.0	35.0	35.0 ± 0.00
	6	28.0	32.0	30.0	30.0 ± 2.00
	7	35.0	35.0	35.0	35.0 ± 0.00
	8	35.0	35.0	35.0	35.0 ± 0.00
	9	33.0	30.0	30.0	31.0 ± 1.73

**Table 4:** Comparison of release percentage at 60 min and 480 min with inhibition zone diameters.

Plate	Release % at 60 min	Release % at 480 min	*S. aureus* Zone (mm)	*E. coli* Zone (mm)	Average Zone (mm)
1	15	99.21	37.0	34.67	35.8
2	29	100.00	35.0	37.00	36.00
3	11	57.27	30.0	31.33	30.8
4	74	100.00	35.0	33.67	34.3
5	36	95.84	32.7	35.00	33.8
6	2	83.05	28.7	30.00	29.3
7	47	100.00	36.3	35.00	35.7
8	0.09	100.00	35.0	35.00	35.00
9	48	94.24	40.0	31.00	35.50

**Table 5:** Two-way ANOVA results for the effects of nozzle speed and distance on drug release. ** indicates statistical significance at *p* < 0.01.

Source	Sum of Squares	df	F	*p*-Value
Speed	9715.20	2	5.60	0.0058 **
Distance	2441.90	2	1.41	0.252
Speed × Distance	2211.21	4	0.64	0.638
Residual	54,660.18	63	-	-

**Figure A1:**
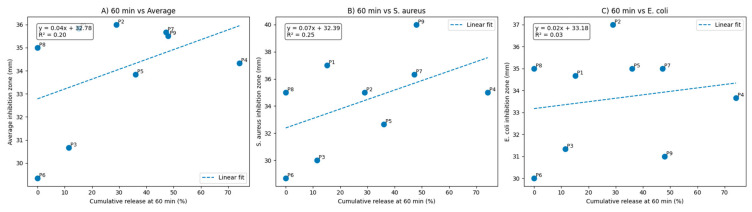
Correlation between cumulative release at 60 min and inhibition zone size. (**A**) Average inhibition zone (mean of *S. aureus* and *E. coli*), (**B**) S. aureus inhibition zone, (**C**) *E. coli* inhibition zone. Dashed lines represent linear regression fits with equations and R^2^ values shown in each panel.

## Data Availability

The data supporting the findings of this study are available from the corresponding author upon reasonable request. Although one of the authors is affiliated with a commercial company, the company had no restrictions on data availability, data interpretation, or publication of the results.

## Appendix A

### Appendix A.1. Assumption Checks

Although the primary analysis was conducted using two-way ANOVA, assumption checks indicated deviations from normality and homogeneity of variance. Shapiro–Wilk tests showed non-normal distributions across speed groups (W = 0.65–0.85, *p* < 0.01), while Levene’s test revealed unequal variances (F(2,65) = 5.73, *p* = 0.005).

### Appendix A.2. Nonparametric Validation

To ensure robustness, nonparametric analyses were performed. The Kruskal-Wallis test confirmed significant differences among speed groups (H = 17.78, df = 2, *p* < 0.001). Pairwise Mann-Whitney U tests with Bonferroni correction showed that 20 cm/s differed significantly from 10 cm/s (*p* < 0.001) and 15 cm/s (*p* = 0.0017), while no difference was observed between 10 and 15 cm/s (*p* = 0.224). These findings are consistent with the ANOVA and Tukey HSD results, supporting the conclusion that nozzle speed is the key determinant of drug release.

**Table A1:** Tukey’s HSD post hoc comparisons for the effect of Speed on drug release.

Comparison (Speed, cm/s)	Mean Difference	95% CI (Lower, Upper)	*p*-Value	Significant (*p* < 0.05)
10 vs. 15	–6.79	–27.06, 13.48	0.703	No
10 vs. 20	–27.33	–47.60, –7.05	0.0053	Yes
15 vs. 20	–20.53	–40.81, –0.26	0.0465	Yes

**Table A2:** Nonparametric tests for the effect of Speed on drug release.

Test	Statistic	df	*p*-Value	Interpretation
Sharipo-Wilk (normality)	W = 0.65–0.85 (per group)	-	*p* < 0.01	Normality violated
Levene’s test (equal variances)	F = 5.73	2.65	0.005	Homogeneity violated
Kruskal-Wallis test	H = 17.78	2	0.00014	Significant differences across groups
Mann-Whitney U (10 vs. 15)	U = 347.5	-	0.224	Not significant
Mann-Whitney U (10 vs. 20)	U = 476.5	-	0.00011	Significant
Mann-Whitney U (15 vs. 20)	U = 441.0	-	0.0017	Significant

### Appendix A.3. Correlation of Early Drug Release with Antimicrobial Activity

To further examine the relationship between early release and antibacterial performance, cumulative release percentages at 60 min were compared with inhibition zone diameters against *S. aureus*, *E. coli*, and their average values. Linear regression analysis indicated weak to moderate correlations, with R^2^ values ranging from 0.03 to 0.25, suggesting that short-term release alone does not fully explain antibacterial activity (Figure A1).

## Appendix B

### Comparative Fitting of Alternative Kinetic Models

AIC values were not calculated for these empirical models; comparison is based on RSS and R^2^.

**Plate**	**Model**	**Equation**	**RSS**	**R^2^**
1	Linear	(0.016x + 4.974)	325.61	0.5788
	Logarithmic	(5.254\ln(x) − 13.413)	112.79	0.8541
	Polynomial	(−4.006 × 10^{−5}x^2 + 0.074x − 0.056)	47.87	0.9381
	Exponential	(25.09(1−e^{−0.0046x}))	40.21	0.9480
2	Linear	(0.009x + 3.782)	101.70	0.5862
	Logarithmic	(3.074\ln(x) − 7.057)	19.82	0.9194
	Polynomial	(−2.292 × 10^{−5}x^2 + 0.042x + 0.904)	10.75	0.9563
	Exponential	(14.96(1−e^{−0.0055x}))	2.30	0.9906
3	Linear	(0.006x + 1.214)	24.96	0.7162
	Logarithmic	(1.757\ln(x) − 4.739)	14.16	0.8390
	Polynomial	(−1.182 × 10^{−5}x^2 + 0.023x − 0.271)	0.78	0.9911
	Exponential	(9.17(1−e^{−0.0030x}))	3.37	0.9617
4	Linear	(0.003x + 3.119)	36.36	0.3427
	Logarithmic	(1.402\ln(x) − 2.079)	8.28	0.8503
	Polynomial	(−1.032 × 10^{−5}x^2 + 0.018x + 1.823)	17.93	0.6759
	Exponential	(6.60(1−e^{−0.0164x}))	5.31	0.9039
5	Linear	(0.007x + 2.607)	43.65	0.6239
	Logarithmic	(2.134\ln(x) − 4.880)	7.13	0.9385
	Polynomial	(−1.476 × 10^{−5}x^2 + 0.028x + 0.753)	5.92	0.9490
	Exponential	(10.46(1−e^{−0.0053x}))	1.69	0.9854
6	Linear	(0.001x + 0.796)	9.55	0.2049
	Logarithmic	(0.502\ln(x) − 1.064)	5.97	0.5027
	Polynomial	(−3.613 × 10^{−6}x^2 + 0.006x + 0.342)	7.29	0.3931
	Exponential	(2.20(1−e^{−0.0114x}))	4.89	0.5926
7	Linear	(0.005x + 3.408)	75.88	0.3312
	Logarithmic	(1.949\ln(x) − 3.798)	22.63	0.8005
	Polynomial	(−1.525 × 10^{−5}x^2 + 0.027x + 1.492)	35.60	0.6862
	Exponential	(8.87(1−e^{−0.0112x}))	11.48	0.8988
8	Linear	(0.003x + 0.600)	11.04	0.6251
	Logarithmic	(0.898\ln(x) − 2.390)	10.16	0.6548
	Polynomial	(−6.636 × 10^{−6}x^2 + 0.013x − 0.234)	3.42	0.8840
	Exponential	(5.06(1−e^{−0.0026x}))	5.21	0.8232
9	Linear	(0.001x + 1.228)	1.99	0.5758
	Logarithmic	(0.383\ln(x) − 0.089)	1.18	0.7482
	Polynomial	(−2.608 × 10^{−6}x^2 + 0.005x + 0.900)	0.81	0.8273
	Exponential	(2.52(1−e^{−0.0115x}))	1.65	0.6478
